# Responses in multiple myeloma should be assigned according to serum, not urine, free light chain measurements

**DOI:** 10.1038/s41375-018-0339-y

**Published:** 2018-12-20

**Authors:** Thomas Dejoie, Jill Corre, Helene Caillon, Philippe Moreau, Michel Attal, Hervé Avet Loiseau

**Affiliations:** 1 Centre Hospitalier Universitaire (CHU), Nantes, France; 2 Institut Universitaire du Cancer, CHU, Centre de Recherche en Cancérologie de Toulouse, INSERM, 1037 Toulouse, France

**Keywords:** Myeloma, Risk factors

## Abstract

The most recent update to the International Myeloma Working Group consensus criteria places a strong emphasis on the need for more sensitive haematological markers of response driven by the success of novel therapies. One such marker is serum free light chain (sFLC) analysis, which was first incorporated into the definition of stringent complete response in 2006. However, over the past decade there has been some reluctance to extend the role of the sFLC assays to replace 24 h urine electrophoresis for monitoring multiple myeloma (MM). In this review, we lay out the evidence in favour of serum over urine for monoclonal FLC measurements and propose modified criteria for response assignment in myeloma.

## Introduction

Monoclonal free light chains (FLCs) in multiple myeloma (MM) patients have historically been monitored in 24 h urine by electrophoretic measurements of Bence Jones protein (BJP), however, much controversy has arisen surrounding the adequacy of this approach for the assessment of FLC response. Limited analytical sensitivity [1, 2], the impact of renal metabolism [3–5], and poor provision of urine samples [6–9] limit the usefulness of urinalysis for monitoring MM patients.

In this context, Freelite^®^ polyclonal immunoassays, which sensitively measure monoclonal serum FLCs (sFLCs) [10], are recommended for the diagnostic work-up of MM [11] and have been included as a biomarker of malignancy in myeloma guidelines [12]. Mounting evidence also suggests that sFLC measurements may be better suited than 24 h urine for monitoring response to therapy. However, except for patients with non-measurable M-protein levels in serum (<10 g/L by SPE) and urine (BJP<200 mg/24 h by UPE), current myeloma guidelines favour urine assessment for monitoring monoclonal FLCs [13–15].

This debate has gathered momentum over the last few years, coinciding with the arrival of clinical and laboratory advances in the early 2000s. Immunomodulatory drugs (IMiDs) and proteasome inhibitors have changed the treatment landscape of MM in recent years by inducing high rates of deep, durable responses [16]. More effective treatments have brought about a clinical need for more sensitive monitoring of monoclonal proteins. Since the last guidelines on serum FLC assessment in MM were published by the International Myeloma Working Group (IMWG) nearly a decade ago [11], a number of publications have addressed the analytical performance and clinical contribution of serum FLC immunoassays over urine analyses in the era of novel agents. Here we discuss the merits of sFLCs measurements for monitoring, and propose modified criteria that incorporate sFLC in place of 24 h urine for response assignment in MM patients.

## Measuring monoclonal FLCs

The concentration of FLCs in serum reflects the balance between rates of production by plasma cells and clearance by the kidneys. Under normal circumstances, FLCs are rapidly removed from serum and metabolised in the proximal tubules of nephrons. Kidneys can metabolise FLCs in quantities far exceeding production, therefore in healthy individuals FLCs are unlikely to be detected in urine by electrophoretic methods. By contrast over 95% intact immunoglobulin MM (IIMM) and, by definition, all light chain MM (LCMM) patients, produce monoclonal FLCs [17, 18]. Large amounts of monoclonal FLCs must be secreted into the serum before the reabsorptive capacity of the tubules is overwhelmed, and BJP can appear in the urine by overflow proteinuria.

Renal metabolism makes 24 h urine BJP measurements in MM patients unreliable [3, 4]; particularly when monoclonal FLC production is low, as typically seen in patients who respond well to treatment. By contrast, Freelite immunoassays quantify FLCs in serum to levels below 1.0 mg/L, hence providing sensitivity many times greater than that of electrophoretic techniques [19]. A direct consequence of renal metabolism is that absolute measurements of FLC in urine and serum show insufficient correlation and cannot be considered interchangeable [18, 20–22]; this has undoubtedly been the main determinant for keeping historic 24 h urine BJP measurements for response assignment in current myeloma guidelines [11].

## Clinical value of serum FLC measurements for monitoring

**Fig. 1:**
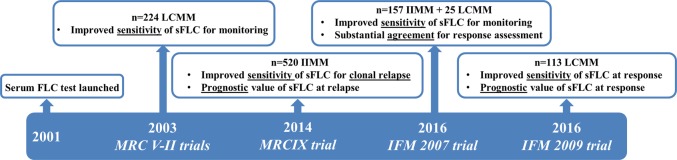
Milestones for serum FLC testing in clinical trials. Year of study publication, number of patients included and clinical impact of serum FLC testing compared to urine assessment are highlighted

Despite insufficient analytical correlation between serum FLC and 24 h urine, many comparative studies have revealed a clinical benefit of serum measurements for monitoring monoclonal FLC, based on three supporting arguments: (1) superior sensitivity for identifying disease; (2) prognostic value during monitoring; and (3) as an early marker of progression (Fig. 1).

### Greater sensitivity of serum FLC measurements for monitoring

As early as 2003, a large study in myeloma patients treated with non-intensive therapy from the UK MRC V–VII trials (1983–1999) reported a far lower rate of LCMM patients achieving a complete response (CR), as determined by normalisation of the sFLC ratio (11%), compared with urinalysis (32%) [18]. Importantly about 10% IIMM patients from the same trial and receiving the same treatment achieved a serological CR, which is comparable to the frequency seen in LCMM using sFLC analysis. These results suggested that 24 h urine measurements lacked sensitivity to reflect tumour burden following therapy, and overestimated the response in a substantial proportion of patients. Analogous results were subsequently reported by the Intergroupe Francophone Du Myélome (IFM) in two independent studies including patients from the IFM2007 [23] and IFM2009 [24] trials who were treated with novel agents. The latter study focused on the responses achieved by patients at the end of VRD (bortezomib, lenalidomide and dexamethasone) induction therapy. At this time, just over 50% of LCMM and IIMM patients had achieved a serological CR as determined by sFLC and SPE assessment, respectively [24]; by contrast 24 h urine was normal in 79% of LCMM patients.

Additionally, trend analyses of patients monitored during treatment indicate that sFLC measurements do not always correlate with urinary BJP measurements over time. In many cases serum measurements are more sensitive and their evolution is consistent with clinical events [5, 23].

### Prognostic value of sFLC during monitoring

Several studies have investigated the association between sFLC measurements during monitoring and patient outcome. The majority demonstrate that normalisation of sFLC levels and ratio after treatment associate with improved outcomes, both in LCMM [25] and IIMM [26] patients.

In newly diagnosed LCMM patients treated with novel therapies, the IFM demonstrated that elevated iFLC levels or an abnormal FLC ratio after induction therapy significantly associated with shorter PFS; by contrast UPE and urine immunofixation (uIFE) had no impact on outcomes [27]. Likewise, in 169 LCMM patients from the GEM/PETHEMA clinical trials, sFLC assays had greater sensitivity than UPE for monitoring low levels of disease in certain cases; and those patients whose FLC ratio remained abnormal or their involved FLC levels elevated after treatment had an increased risk of progression [28].

Paiva was the first to show, in a small study of elderly (>65 years), non-transplant eligible patients, that those achieving a stringent CR (sCR) and an immunophenotypic response (determined by flow cytometry) had superior PFS outcomes than those with an immunophenotypic response but not in sCR; although statistical significance was not reached, possibly owing to small sample size and an unconventional categorisation of sCR patients [29]. Subsequently Kapoor assessed the prognostic value of sCR in a prospective study of a combined 445 LCMM and IIMM patients undergoing autologous stem cell transplantation. Patients attaining sCR post-transplant had a distinctive survival advantage over those achieving a CR [26]; hence supporting the inclusion of sCR as a response category in IMWG guidelines [14, 15]. In a later report, Moustafa showed that normalisation of the sFLC ratio retains its prognostic significance throughout monitoring, independent of depth of response [30].

### sFLCs as biomarkers of relapse

Serum FLC assays have proven useful markers of progressive disease, and may identify relapse earlier than traditional methods including urine [5, 31, 32]. In IIMM patients who relapse early after successful treatment, the short half-life of sFLCs offer a distinctive advantage over serum IFE for detecting progression, particularly in IgG MM patients [33]. Direct comparisons between serum FLC and 24 h urine also indicate that the former have greater sensitivity for detecting residual disease preceding clinical relapse [34] and for identifying light chain escape [35–37].

## Serum FLCs for response assignment

Dispenzieri appraised serum and urine FLC measurements as markers of response, and demonstrated the lack of correlation between methods after just two treatment cycles [22]. The study also showed that early sFLC responses predicted for eventual overall response; however, there was no association with progression-free survival (PFS) and overall survival (OS). Dejoie also reported discrepancies between sFLC and 24 h urine assessment after three treatment cycles, but in this case sFLC measurements associated with both PFS and OS [27]. Fundamental differences between these studies were in patient selection and the induction protocol, which in Dispenzieri included all MM patients treated with obsolete therapy (VBMCP; vincristine, carmustine, melphalan, cyclophosphamide, and prednisone), whereas for Dejoie was restricted to LCMM patients undergoing induction with novel agents (VRD).

A limitation of the latter study was the lack of bone marrow data at the end of induction, which prevented the authors from determining how many patients with negative urine IFE were in CR. Since normalisation of urine at this stage did not associate with improved outcomes, it seems reasonable to hypothesise that 24 h urine measurements were not reflective of bone marrow plasma cell (BMPC) content, for BMPC < 5% post-induction with novel agents has been reported to associate with longer PFS and OS, at least in the transplant setting [38, 39]. By contrast, normalisation of sFLC parameters in the same study translated into superior PFS, OS and as predictors of immunophenotypic response, substantiating the argument that early responses based on sFLC changes are possibly more representative of tumour response.

Accurate monitoring shortly after initiation of treatment has become an ever growing clinical necessity in the era of novel drugs, with potential economic implications. Deeper responses during induction therapy with bortezomib-containing regimens are associated with improved outcomes in newly diagnosed patients [40, 41]. Conversely, failure to achieve early haematological response translates into inferior survival. Based on these distinct outcomes, a recent report advocated the inclusion of patient stratification in future MM trial design based on quality of response during induction therapy. The observation that poor responders may benefit from early dose-escalation is particularly relevant in countries with restricted funding for novel agents [42]. In this context, the better sensitivity of sFLC over 24 h urine measurements may offer more accurate monitoring conducive to cost-effective treatment decisions [43, 44].

The impact of replacing 24 h urine for serum FLC measurements has also been investigated at maximum response. In a cohort of 25 LCMM and 157 IIMM patients from the IFM2007 MM trial [23], responses based on serum methods (SPE + sFLC) demonstrated near-perfect agreement to standard SPE + uIFE assessment by IMWG guidelines. The main limitations of this and other comparative studies have been the lack of bone marrow samples to ascertain CR and the absence of results correlating response and clinical outcome. In an unpublished study including 450 IIMM patients from the IFM2009 trial, responses were assigned post-consolidation and post-maintenance therapy using IMWG response criteria; or modified criteria replacing 24 h urine for serum FLC assessment. As in previous reports, serum FLC measurements in this cohort were more sensitive than urines for identifying disease; however response assignment by both criteria, and PFS outcomes based on response, were comparable.

## Conclusions

Although urine testing can provide useful information of underlying renal pathology due to glomerular or tubular dysfunction, and for screening patients with suspected AL amyloidosis [11, 45], there are many drawbacks to the use of 24 h urine BJP measurement for monitoring myeloma. Practical considerations include the difficulties to obtain complete 24 h collections from MM patients, who are often elderly and frail; the lack of standard protocols for the concentration of urine specimens; and poor compliance in the provision of a urine sample. However, renal physiology remains the most important issue concerning the use of urine for monitoring FLCs. Renal metabolism causes in most circumstances urinalysis to be an insensitive test for measuring monoclonal light chains, being primarily responsible for the lack of correlation with serum tests.

Replacing 24 h urine for sFLC measurements for monitoring IIMM patients results in equivalent response assignment, which in these patients relies mostly on M-protein changes as determined by SPE. This complements previous observations demonstrating clinical superiority of serum compared to urine assessment for monitoring LCMM patients. There is now cumulative and substantial evidence supporting the clinical and practical benefit of sFLC measurements for monitoring myeloma. We suggest the inclusion of sFLC to replace 24 h urine for the assignment of response in all MM patients, and propose modified response criteria modelled on those currently recommended by the IMWG (Table 1). Such criteria are already in place for monitoring oligosecretory patients, therefore our proposal would help to unify, and consequently simplify, current response criteria, making it pertinent to a greater proportion of patients.

**Table 1:** IMWG and proposed modified response criteria for multiple myeloma

Response category	IMWG response criteria^a^	Proposed modified response criteria^a^
Stringent complete response (sCR)	CR as defined below plus:	CR as defined below plus:
	• Normal FLC ratio and	• Absence of clonal cells in bone marrow by immunohistochemistry or 2–4-colour flow cytometry
	• Absence of clonal cells in bone marrow by immunohistochemistry or 2–4-colour flow cytometry	
Complete response (CR)	• Negative immunofixation on the serum and urine and	• Negative immunofixation on the serum and
	• Disappearance of any soft tissue plasmacytomas and	• Normal FLC ratio and
	• ≤5% plasma cells in bone marrow	• Disappearance of any soft tissue plasmacytomas and
		• ≤5% plasma cells in bone marrow
Very good partial response (VGPR)	• Serum and urine M-protein detectable by immunofixation but not on electrophoresis or ≥90% reduction in serum M-protein plus urine M-protein level < 100 mg per 24 h	• Serum M-protein detectable by immunofixation but not on electrophoresis or ≥90% reduction in serum M-protein and,
		• Abnormal FLC ratio and ≥90% reduction in the difference between involved and uninvolved FLC (dFLC) levels
Partial response (PR)	• ≥50% reduction of serum M-protein and	• ≥50% reduction of serum M-protein and
	• reduction in 24-h urinary M-protein by ≥90% or to <200 mg per 24 h^b^	• Abnormal FLC ratio and ≥50% reduction the difference between involved and uninvolved FLC (dFLC) levels^c^
Stable disease (SD)	• Not meeting criteria for CR, VGPR, PR or progressive disease	• Not meeting criteria for CR, VGPR, PR or progressive disease
Progressive disease (PD)	Requires any one or more of the following:	Requires any one or more of the following:
	• Increase of ≥25% from lowest response in serum M-component (the absolute increase must be ≥5 g/L) and/or urine M-component (the absolute increase must be ≥200 mg/24 h).	• Increase of ≥25% from lowest response in serum M-component (the absolute increase must be ≥5 g/L) and/or the difference between involved and uninvolved FLC (dFLC) levels. The absolute increase must be >100 mg/L.
	• Only in patients without measurable serum and urine M-protein levels: the difference between involved and uninvolved FLC (dFLC) levels. The absolute increase must be >100 mg/L.	• Bone marrow plasma cell percentage: the absolute % must be ≥10%
	• Only in patients without measurable serum and urine M-protein levels and without measurable disease by FLC levels, bone marrow PC percentage (absolute percentage must be ≥10%)	• Definitive development of new bone lesions or soft tissue plasmacytomas or definite increase in the size of existing bone lesions or soft tissue plasmacytomas
	• Definitive development of new bone lesions or soft tissue plasmacytomas or definite increase in the size of existing bone lesions or soft tissue plasmacytomas	• Development of hypercalcaemia (corrected serum calcium >115 mg/L or 2.65 mmol/L) that can be attributed solely to the plasma cell proliferative disorder
	• Development of hypercalcaemia (corrected serum calcium >115 mg/L or 2.65 mmol/L) that can be attributed solely to the plasma cell proliferative disorder	

^a^All response categories require two consecutive assessments made at any time before the institution of any new therapy; all categories also require no known evidence of progressive or new bone lesions if radiographic studies were performed. Radiographic studies are not required to satisfy these response requirements. VGPR and CR categories require serum studies (and urine for IMWG criteria) regardless of whether disease at baseline was measurable on serum, urine, both, or neither.

^b^If the serum and urine M-protein are unmeasurable, ≥50% reduction in plasma cells is required, provided baseline bone marrow plasma cell percentage was 30%.

^c^If the serum FLC is unmeasurable, ≥50% reduction in plasma cells is required, provided baseline bone marrow plasma cell percentage was 30%. Criteria for coding PD: Bone marrow criteria for PD are to be used only in patients without measurable disease by M protein and by FLC levels; “25% increase” refers to M protein, FLC, and bone marrow results, and does not refer to bone lesions, soft tissue plasmacytomas, or hypercalcemia and the “lowest response value” does not need to be a confirmed value
